# A novel frameshift *ACTN2* variant causes a rare adult‐onset distal myopathy with multi‐minicores

**DOI:** 10.1111/cns.13697

**Published:** 2021-06-25

**Authors:** Lei Chen, Dian‐Fu Chen, Hai‐Lin Dong, Gong‐Lu Liu, Zhi‐Ying Wu

**Affiliations:** ^1^ Department of Neurology and Research Center of Neurology in Second Affiliated Hospital, and Key Laboratory of Medical Neurobiology of Zhejiang Province Zhejiang University School of Medicine Hangzhou China

**Keywords:** *ACTN2*, aggregates, Chinese family, distal myopathy, multi‐minicores

## Abstract

**Introduction:**

Distal myopathies are a group of rare muscle disorders characterized by selective or predominant weakness in the feet and/or hands. In 2019, *ACTN2* gene was firstly identified to be a cause of a new adult‐onset distal muscular dystrophy calling actininopathy and another distinctly different myopathy, named multiple structured core disease (MsCD). Thus, the various phenotypes and limited mutations in ACTN2‐related myopathy make the genotype‐phenotype correlation hard to understand.

**Aims:**

To investigate the clinical features and histological findings in a Chinese family with distal myopathy. Whole exome sequencing and several functional studies were performed to explore the pathogenesis of the disease.

**Results:**

We firstly identified a novel frameshift variant (c.2504delT, p.Phe835Serfs*66) within *ACTN2* in a family including three patients. The patients exhibited adult‐onset distal myopathy with multi‐minicores, which, interestingly, was more like a combination of MsCD and actininopathy. Moreover, functional analysis using muscle samples revealed that the variant significantly increased the expression level of α‐actinin‐2 and resulted in abnormal Z‐line organization of muscle fiber. Vitro studies suggested aggregate formations might be involved in the pathogenesis of the disease.

**Conclusion:**

Our results expanded the phenotypes of ACTN2‐related myopathy and provided helpful information to clarify the molecular mechanisms.

## Graphical abstract

A rare adult‐onset distal myopathy with multi‐minicores caused by a novel *ACTN2* mutation. Functional analysis using muscle samples revealed that the variant significantly increased the expression level of α‐actinin‐2 and resulted in abnormal Z‐line organization of muscle fiber. Vitro studies suggested aggregate formations might be involved in the pathogenesis of the disease.

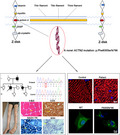

## 1 INTRODUCTION

Distal myopathies are a group of rare muscle disorders characterized by selective or predominant weakness in the feet and/or hands.^1^ The age at onset can be variable ranging from early childhood to even late adulthood. The myopathological findings are also heterogeneous and vary widely including dystrophic changes, rimmed vacuoles, and myofibrillar features. To date, more than 25 different genes have been identified, exhibiting autosomal dominant or recessive inheritance pattern.^1^, ^2^ Notably, a significant number of the identified genes encode proteins that are component of Z‐disk or Z‐disk relevant structures such as *ZASP*,^3^ *DES*,^4^ *MYOT*,^5^, ^6^ *FLNC*,^7^ and *TTN*.^8^

Alpha‐actinin‐2 encoded by *ACTN2* gene is one of the major components of the Z‐disk.^9^ It crosslinks actin filaments and sarcomeric proteins such as titin^10^, ^11^ and myopalladin^12^ to maintain the integrity of the contractile apparatus. The gene *ACTN2* was previously known as a causative gene of cardiomyopathies.^13^, ^14^ In 2019, two distinctly different skeletal muscle diseases including multiple structured core disease (MsCD) in congenital myopathy and adult‐onset distal muscular dystrophy calling actininopathy were reported caused by *ACTN2* mutations.^15^, ^16^

Although myofibrillar features are the most common characteristics in various distal myopathies caused by genes encode Z‐disk or Z‐disk relevant protein, it seems that no aggregates are found in muscle biopsy of ACTN2‐related myopathy. Instead, in MsCD, multiple structured cores are the main histological findings. Core pathology (central cores and multiple minicores) is rare in adult‐onset distal myopathy. It has been reported that multicore‐like unevenness of stain could occasionally occur in patients with distal myopathy caused by *RYR1* mutations.^17^, ^18^

Herein, we firstly identified a novel *ACTN2* variant causing an adult‐onset distal myopathy with a morphological feature of multiple well‐defined minicores, which, interestingly, was more like a combination of MsCD and actininopathy. Further, we investigated the functional characterization of this mutation, together with the previously reported mutations in ACTN2‐related myopathy, providing insightful information to clarify the molecular mechanisms underlying.

## 2 MATERIALS AND METHODS

### 2.1 Study subjects and clinical examinations

Three patients and four family members from a Chinese pedigree affected by distal myopathy were collected in December 2019. The clinical evaluations and neurological examinations were performed by two senior neurologists. The patients were diagnosed with distal myopathy based on the clinical features and myopathological results. This study was approved by the Ethics Committee of the Second Affiliated Hospital, Zhejiang University School of Medicine. Written informed consents were obtained from all the participants.

### 2.2 Immunohistochemical analysis

Open muscle biopsy of the quadriceps was obtained from the proband at the age of 46. Standard histochemical reactions on 8 µm frozen sections encompassed hematoxylin and eosin (H&E), modified Gomori trichrome (MGT), nicotinamide adenosine dinucleotide‐tetrazolium reductase (NADH‐TR), succinate dehydrogenase (SDH), cytochrome C oxidase (COX), and the adenosine triphosphatase (ATPase pH = 4.3, 4.6, and 9.4, respectively) reactions.

### 2.3 Genetic analysis

Genomic DNA was extracted from peripheral EDTA‐treated blood using a commercial blood genomic extraction kit (Qiagen, Hilden). Whole exome sequencing (WES) was performed on the Illumina HiSeq X Ten platform (XY Biotechnology Co. Ltd.), captured by the Agilent Sure Select Human All Exon V6 products. For details on sequencing protocols, bioinformatic analysis and filtering methods can be found in our previously reported publications.^19^, ^20^, ^21^ All variants were filtered with the frequency ≤0.05 in Genome Aggregation Database (gnomAD) (http://gnomad.broadinstitute.org/), ExAC database (ExAC) (https://exac.broadinstitute.org/), and 1000 Genomes Project (https://www.ncbi.nlm.nih.gov/variation/tools/1000genomes/), and variants that were not in the exonic or splicing site were excluded. Three software programs, including SIFT (http://sift.jcvi.org/), PolyPhen‐2 (http://genetics.bwh.harvard.edu/pph2/), and MutationTaster (http://www.mutationtaster.org/), were used to predict the possible changes in protein function caused by the variants. Sanger sequencing was performed to confirm the potential variants and co‐segregation of the pedigree.

### 2.4 Immunoblotting analysis

Frozen muscle samples of the proband and two controls with normal muscle pathology were resolved by 10% SDS‐polyacrylamide gel electrophoresis (SDS‐PAGE), transferred to polyvinylidene fluoride (PVDF) membranes. The following primary antibodies were used to detect the specific bands: anti‐ACTN2 (A8939; Abclonal), anti‐ACTA1 (A2172; Sigma‐Aldrich), and anti‐GAPDH (8884S; Cell Signaling Technology).

### 2.5 Plasmid constructs and reagents

The wild‐type (WT) full‐length coding region of human *ACTN2* (NM_001103) with additional 200bp 3’UTR nucleotides was cloned into pcDNA.3.1‐3Xflag‐C and pEGFP‐C2 vectors (Invitrogen). All the mutant constructs of *ACTN2*, including c.2504delT (p. Phe835Serfs*66) in present study and three previously reported mutations, c.392T>C (p. Leu131Pro), c.1459T>C (p. Cys487Arg), and c.2180T>G (p. Leu727Arg), were created by PCR mutagenesis and verified by Sanger sequencing. They were designated as pcDNA3.1‐Flag‐WT (Flag‐WT) and pEGFP‐WT (eGFP‐WT); pcDNA3.1‐Flag‐Phe835Serfs*66 (Flag‐F835Sfs*66) and pEGFP‐Phe835Serfs*66 (eGFP‐F835Sfs*66); pcDNA3.1‐Flag‐Leu131Pro (Flag‐L131P) and pEGFP‐Leu131Pro (eGFP‐L131P); pcDNA3.1‐Flag‐Cys487Arg (Flag‐C487R) and pEGFP‐Cys487Arg (eGFP‐C487R); pcDNA3.1‐Flag‐Leu727Arg (Flag‐L727R) and pEGFP‐Leu727Arg (eGFP‐L727R), respectively.

### 2.6 Cell culture and transfection

HEK‐293T and C2C12 cells were maintained at 37℃ in Dulbecco's modified Eagle's medium (DMEM, HyClone) supplemented with 10% fetal bovine serum (FBS, Gibco). HEK‐293T cells were transfected with expression vectors with Lipofectamine 3000 (Invitrogen) and C2C12 myoblasts with TurboFect (Thermo Fisher Scientific) according to the manufacturer's instructions.

### 2.7 Immunofluorescence analysis

Frozen muscle sections were fixed at room temperature in acetone for 10 min and washed in PBS. Nonspecific sites were blocked in PBS with 1% BSA and 0.01% Triton X‐100 for 1 h and incubated overnight at 4°C with rabbit anti‐ACTN2 (A8939; Abclonal). After washing, the sections were incubated 1h with the secondary antibody: Alexa Fluor 594 goat anti‐rabbit antibodies at room temperature. Cell nuclei were then stained with 40, 6‐diamidino‐2‐phenylindole (DAPI; Life Technologies). For C2C12 cell lines, after 24 h transfection with eGFP‐tagged ACTN2, cells were fixed at room temperature in 4% PFA for 10 min. DAPI was used for the staining of cell nuclei. Fluorescence images were captured by Olympus FV3000 OSR confocal system.

### 2.8 Protein solubility assay

Cells were pelleted and lysed with the lysis buffer (CelLytic™ M, Sigma) on ice for 20 min. Subsequent centrifugation at 12,000 rpm for 15min at 4°C resulted in a soluble and insoluble fraction. The insoluble fraction was resuspended in the following lysis buffer (10% SDS and 1 M Tris‐buffer with protease inhibitors) and sonicated with 10 × 1‐s bursts at 10% amplitude and 0.6 pulse rate. Western blotting proceeded as described previously. Specific bands were detected by the following antibodies: anti‐Flag (14793S; Cell Signaling Technology) and anti‐β‐Tubulin (5346S; Cell Signaling Technology).

### 2.9 Statistical analysis

GraphPad Prism 8.0 and Adobe Photoshop CC 2017 software were used for data analysis. Data were first tested by Shapiro‐Wilk test for normality and lognormality. All the experiments were repeated at least three times independently, and data were expressed as the mean ±standard deviation. One‐way ANOVA test was performed followed by Turkey's multiple comparisons test for multiple comparisons. *p* value <0.05 was considered statistically significant.

## 3 RESULTS

### 3.1 Clinical features

**FIGURE 1:**
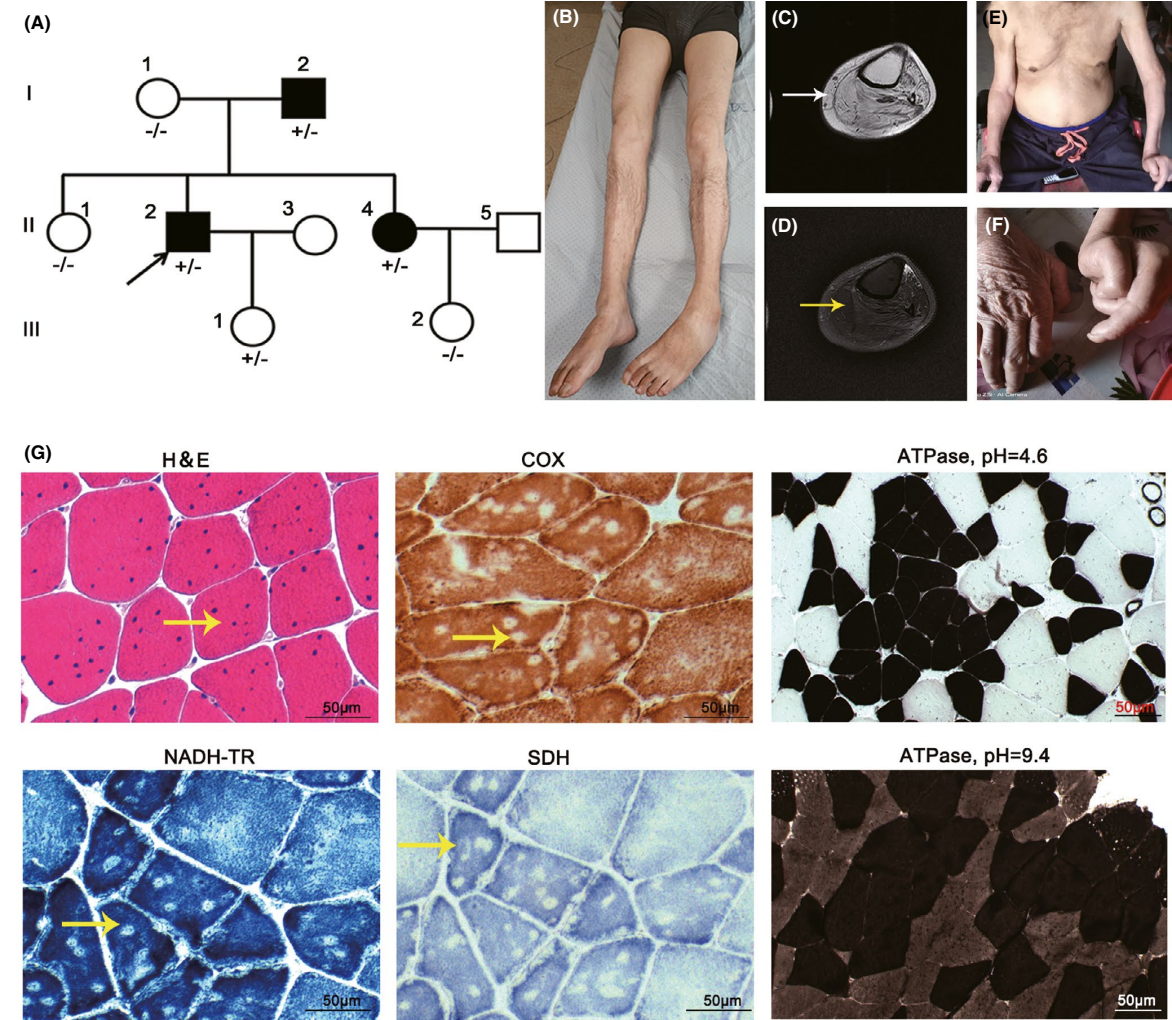
Clinical and histopathological features in our patients. (A) Pedigree of our family with *ACTN2* variant. Circle: females; square: males; open symbol: unaffected; filled symbol: affected; arrow: proband. The “+” symbols indicate the presence and the “‐” symbols indicate the absence of the mutation. (B) Marked atrophy of the proband's legs. (C and D) MRI imaging of the proband. Extensive fatty infiltration (white arrow) and partial edema (yellow arrow) on the left leg. (E) Severe upper limbs atrophy in the father (I‐2). (F) Contractures in hands of the father (I‐2). (G) Histological findings in the proband. Internal nuclei (yellow arrow) in H&E staining. Multiple well‐delimited cores (yellow arrow) observed in NADH‐TR, SDH, and COX reactions. ATPase reaction (pH =4.6 and 9.4, respectively) showed both type I and type II fibers‐grouping and atrophy predominantly in type I fibers; Scale bar, 50 μm

The proband of the family (Figure 1A) is a 46‐year‐old male who had symmetric weakness of distal lower limbs at the age of 39. As the disease slowly progressed, he developed foot drop and noticed muscle atrophy in his distal legs. One year ago, he complained of chest tightness when walking for a long time and progressed to heart failure. Echocardiography at that time revealed decreased left ventricular diastolic function. On examination, he had bilateral steppage gait, severe distal lower limb atrophy, and weakness with Medical Research Council (MRC) grade 2/5 and mild proximal weakness (MRC 4/5) (Figure 1B). Neck flexion strength was reduced (MRC 3/5), and reflexes were absent in the lower limbs. Sensation was intact. The creatine kinase (CK) level was normal with 85 U/L (reference <194 U/L), and EMG demonstrated positive sharp waves and fibrillation potentials, accompanied by short‐duration, polyphasic, and early recruited motor unit potentials (MUPs). Muscle magnetic resonance imaging (MRI) of his left leg revealed extensive fatty degeneration and muscle edema (Figure 1C‐D). Echocardiography showed no significant abnormalities.

His father (I‐2) presented with lower limb weakness at the age of 40. In addition to distal lower limbs weakness, he developed severe weakness of proximal upper limbs and contractures of his hands (Figure 1E‐F). He lost his ambulation at the age of 65. Besides, he also had a history of heart failure. Muscle atrophy was marked in the proximal upper limbs and not obvious in his lower limbs. The CK level was normal with 25 U/L (reference <194 U/L). His 38‐year‐old younger sister (II‐3) had asymmetrical muscle involvements in the distal lower limbs for about 2 years. On examination, she had asymmetric bilateral ankle dorsiflexion weakness (MRC 3/5 in right and MRC 4/5 in left) with steppage gait. EMG showed low‐amplitude, polyphasic MUPs with spontaneous potential in the distal leg muscles. There is no symptom in his 19‐year‐old daughter whose CK level was normal with 77 U/L (reference <170 U/L).

### 3.2 Muscle pathology

Quadriceps muscle biopsy was performed in the proband. H&E staining revealed fiber size variability with remarkable internal nuclei. Oxidative enzyme reactions uncovered several small well‐delimited cores, predominantly in type I fibers. ATPase reaction showed both type I and type II fibers‐grouping (Figure 1G). No necrotic fibers were observed, and no aggregates or nemaline rods were found in the histopathologic staining.

### 3.3 Genetic analysis and the spectrum of ACTN2 mutations

**FIGURE 2:**
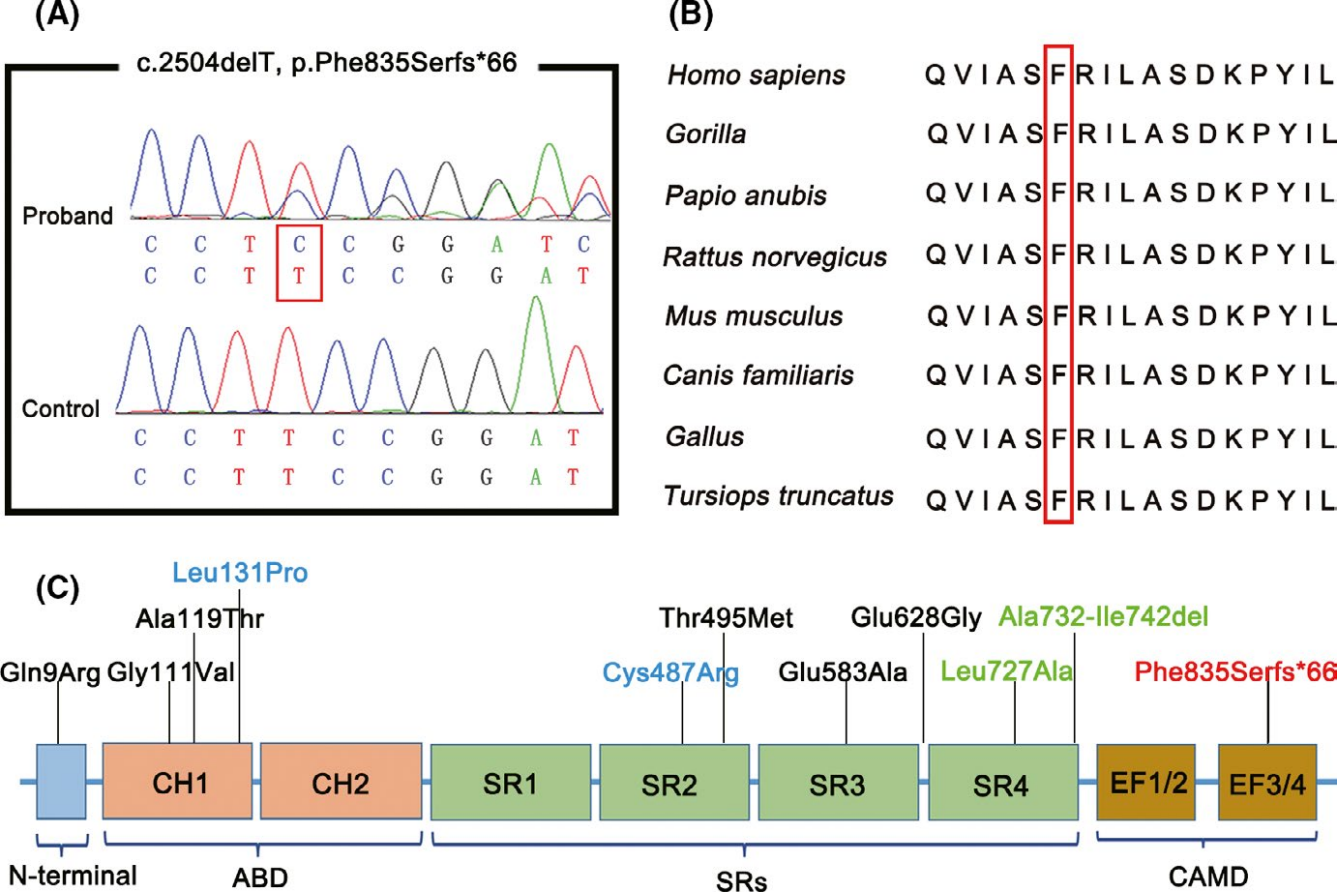
Genetic findings in patients and pathogenic variants in *ACTN2*. (A) Sequencing chromatogram of the presence of mutation c.2504delT, p. Phe835Serfs*66 in the proband (upper), and absence in the control (lower). (B) The Phe835 residue is highly conserved in different species. (C) Schematic representation of *ACTN2* and summary of reported pathogenic variants. Red: novel variants; Blue: actininopathy‐related variants; Green: MsCD‐related variants; Black: HCM, DCM and/or other cardiac abnormalities‐related variants

After WES analysis on the proband, a novel heterozygous frameshift variant (c.2504delT, p. Phe835Serfs*66) in *ACTN2* (NM_001103.3) was identified and confirmed by Sanger sequencing (Figure 2A). The co‐segregation was demonstrated. The variant was absent in the gnomAD, ExAc, and 1000 genomes databases and located at evolutionarily highly conserved region (Figure 2B). Moreover, the single nucleotide deletion prolonged the original stop codon, resulting in a C‐terminal extension of the protein. According to the standard of American College of Medical Genetics and Genomics (ACMG),^22^ the variant was classified as a pathogenic variant.

To date, a lot of heterozygous pathogenic variants have been reported related to cardiomyopathies and/or other cardiac abnormalities, while, only 4 variants were associated with skeletal myopathies (HGMD https://my.qiagendigitalinsights.com/bbp/view/hgmd/pro/start.php). These variants were widely distributed, and most were found in the SRs domain, followed by the ABD domain (Figure 2C).

### 3.4 The analysis of α‐actinin‐2 expression and distribution

**FIGURE 3:**
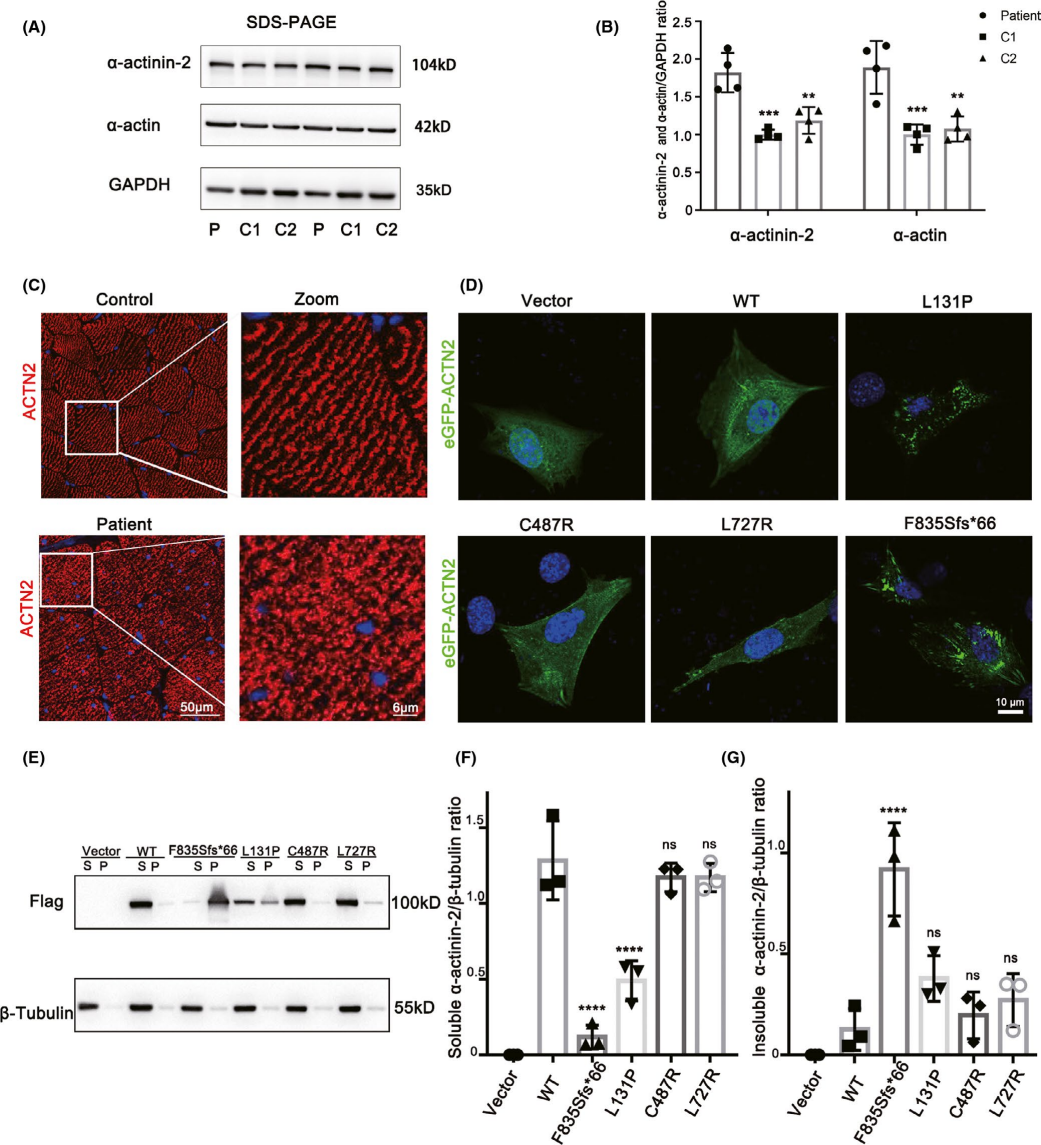
Functional analyses of *ACTN2* variants. (A) Western blot analysis of α‐actinin‐2 and α‐actin expression in protein extracts obtained from the proband's muscle. (B) The graph shows GAPDH normalized α‐actinin‐2/α‐actin expression. (*n* = 4 biological repeats, data are shown as mean ±SD, **p < 0.01; ***p < 0.001). (C) Alpha‐actinin‐2 (red) immunofluorescence analysis. Scale bar, 50 μm. (D) Alpha‐actinin‐2 localization in C2C12 myoblasts expressing WT or mutant eGFP‐ACTN2. Scale bar, 10 μm. (E) Soluble (S) and insoluble (P) protein fraction per variant with Western blot probing for Flag‐ACTN2 and β‐Tubulin. F‐G, The graphs show β‐Tubulin normalized Flag‐α‐actinin‐2 level per variant in soluble protein fraction (F) and insoluble protein fraction (G). (*n* = 3 biological repeats, data are shown as mean ± SD. ****p < 0.0001; ns: not significantly)

To assess the impacts on the α‐actinin‐2 expression by the identified variant, we performed Western blot analysis of the muscle extracts obtained from the proband and two controls with normal pathology. The results revealed significantly increased level of α‐actinin‐2 and α‐actinin in the proband compared with the controls (Figure 3A‐B). Moreover, immunofluorescence analysis on muscle frozen sections showed a structural turbulence of α‐actinin‐2 staining in the proband compared with the control who exhibited a highly ordered structure (Figure 3C), indicating the abnormal Z‐disk organization.

### 3.5 Protein subcellular localization

To further elucidate the biological effects, the plasmids with N‐terminal eGFP or flag tagged WT, F835Sfs*66, and three previously reported variants including L131P (actininopathy), C487R (actininopathy), and L727R (MsCD) were constructed. We found diffused intracellular α‐actinin‐2 in C2C12 myoblasts transiently expressing eGFP‐WT, eGFP‐C487R, and eGFP‐L727R. By contrast, the eGFP‐F835Sfs*66 and eGFP‐L131P mutants displayed aggregates throughout the cytosol (Figure 3D). Then, we investigated the presence of α‐actinin‐2 aggregations in the soluble and insoluble fraction by Western blot. In the HEK‐293T cells transfected with Flag‐WT, Flag‐C487R, and Flag‐L727R, α‐actinin‐2 was mainly present in the soluble fraction; however, in cells transfected with Flag‐F835Sfs*66 and Flag‐L131P, we detected an enrichment of α‐actinin‐2 in the insoluble fraction which was consistent with the observation in C2C12 myoblasts (Figure 3E‐G). Altogether, the data confirmed the p. Phe835Serfs*66 and p. Leu131Pro resulted in aggregates of α‐actinin‐2.

## 4 DISCUSSION

In this study, we firstly reported a novel heterozygous pathogenic variant within *ACTN2* causing distal myopathy with multi‐minicores in a Chinese family. The variant resulted in an increased expression level of α‐actinin‐2, abnormal Z‐disk organization, and aggregate formations, implying the disease pathogenesis, might relate to aggregate formations.

The presence of minicores with the loss of oxidative enzyme activity on muscle biopsy in our patient suggested the diagnosis of minicore disease.^23^ Core myopathies including central core disease (CCD) and multi‐minicore disease (MmD) are often considered preeminently pediatric conditions characterized by marked extensive weakness, early spinal rigidity, and respiratory impairment.^24^ The most common causative gene for core myopathies is *RYR1* and less frequent one is *SEPN1*. An unusual adult‐onset, mild calf‐predominant myopathy in combination with well‐defined cores and multicore‐like unevenness of stain was reported due to *RYR1* mutations (Table 1). In addition, a sporadic case with a missense *RYR1* mutation was reported to exhibit an adult‐onset, anterior tibial muscle‐prominent distal myopathy with multiple cores pathology.^17^ Compared to our patients, they showed mild lower limb distal muscle weakness and no upper limbs weakness and contractures were observed.

**TABLE 1:** Clinical features and genetic findings of the patients with actininopathy, MsCD, and calf‐predominant myopathy

	Actininopathy	MsCD	Calf‐predominant myopathy
Inheritance	AD	unknown	AD
AAO (Mean±SD; Median)	42.87 ± 10.6; 41	Neonatal or early childhood	30.44 ± 15.6; 40
Initial symptoms	Foot drop/calf pain/asymptomatic/quadriceps atrophy/extensor carpi radialis atrophy	Hypotonia and sucking difficulties/LL weakness	Ankle weakness/ asymptomatic/ myalgias
Muscle affected	LL lower limbs, progressive to UL weakness	Diffuse muscle atrophy; bilateral ptosis, ophthalmoparesis; mild facial weakness	Mild calf‐predominant weakness; mild proximal weakness (uncommon)
Cardiac involvement	Ventricular hypertrophy and atrial flutter (uncommon)	None	None
Deformations	None	Dorsal kyphoscoliosis; contractures of joints; spinal rigidity; mild thoracic scoliosis	Achilles tendon tightness
CK	Ranged widely: normal to elevated >10×	Normal	Ranged widely: 1.5×~10×
Muscle pathology	Fiber size variability, centralized nuclei; rimmed vacuole	Fiber size variability; centralized nuclei; multiple structured minicores	Internal nuclei; marked fiber size variation; well‐defined cores and multicore‐like unevenness of stain
Gene	*ACTN2*	*ACTN2*	*RYR1*
Reference	^16^	^15^	^18^

Abbreviation: AAO, age at onset; CK, creatine kinase; LL, lower limbs; UL, upper limbs.

Patients with *ACTN2* mutations could present with a wide clinical spectrum including cardiomyopathies and/or other cardiac abnormalities, MsCD, and actininopathy. MsCD is characterized by progressive early‐onset extensive myopathy clinically manifesting with severe muscle atrophy, facial weakness, contractures, and respiratory symptoms and a unique histopathology of multiple structured cores in the biopsy, and actininopathy is a late‐onset symmetric/asymmetric distal muscular dystrophy (Table 1). In this study, our patient presented with an adult‐onset distal myopathy with multi‐minicores, progressive to proximal upper and lower limbs with contractures, which, interestingly, was more like a combination of MsCD and actininopathy. Besides, cardiomyopathies were not remarkable in all reported patients with ACTN2‐related myopathy, but heart failure or cardiac arrhythmia could be occurred in some patients.^16^

Alpha‐actinin‐2 is composed of an N‐terminal actin‐binding domain (ABD), a central rod domain of four spectrin‐like repeats (SRs), and a C‐terminal calmodulin‐like domain (CAMD) with two EF‐hand motifs (EF1/2 and EF3/4).^11^ It functionally acts as a homodimer where SRs repeats are thought to be responsible for the dimerization.^25^ The EF3/4 domain interacts with the “neck” region between the ABD and first SR of the opposing monomer, making actinin to be a “closed” conformation.^10^, ^11^ This closed conformation will be changed into an “opening” structure when phospholipid binding to the ABD, allowing the interactions of α‐actinin‐2 with actin filaments as well as the titin Z‐repeats.^10^, ^26^ Such conformational switch provides its structural rigidity and plasticity, essential for the maintenance of integrity in contractile apparatus during muscle contraction. The present mutation p. Phe835Serfs*66 located in the EF3/4 domain was close to Ser834 site, which was thought to be an important EF3/4‐neck‐interacted position.^11^ Thus, we hypothesized that the present mutation might affect the EF3/4‐neck interaction, resulting in abnormal binding of α‐actinin‐2 with actin and titin, leading to an impaired conformational change and eventually disruption of sarcomere.

The increased expression level of α‐actinin‐2 and α‐actin, Z‐line disorganization and aggregate formations supported the assumption in some degree. Moreover, in cells transfected with the p. Leu131Pro located in ABD domain, we observed the similar aggregations, suggesting the notion that the impaired conformational change possibly participated in the pathogenesis of p. Phe835Serfs*66 and p. Leu131Pro. Since SRs domain acts as an interface of multiple interactions with various proteins, it possibly explains for the distinguished clinical heterogeneity caused by variants in SRs domain. Besides, it is noteworthy that genes encoded the Z‐disk or Z‐disk relevant proteins could cause different myopathies. For example, mutations in gene *ACTA1* encoding α‐actin cause 9 different muscle pathologies such as actin filament aggregates, core‐like areas, nemaline bodies et al.,^27^ and various of titinopathy due to *TTN* variants.^28^ Thus, the multiple interactions of proteins in the Z‐disk possibly account for the various phenotypes. Therefore, additional patients with ACTN2‐related myopathy are important to investigate the clinical features and more functional studies are needed to clarify the molecular mechanisms. Since most core diseases are caused by *RYR1* variants, it may also be another target in excitation‐contraction coupling for the studies of ACTN2‐related myopathy.^24^, ^29^

In conclusion, we identified a novel *ACTN2* pathogenic variant as the cause of adult‐onset distal myopathy with multi‐minicores in a Chinese family. Functional studies indicated that the abnormal protein aggregates possibly participated in the pathogenesis and eventually resulted in structural turbulence of Z‐line. This study expanded the clinical and spectrum of *ACTN2* variants and provided a perspective to understand the various clinical phenotypes and complex mechanisms caused by *ACTN2* mutations.

## CONFLICT OF INTEREST

The authors declare no conflict of interest.

## Supporting information

Supplementary Material

## Data Availability

The data that support the findings of this study are available from the corresponding author upon reasonable request.

## References

[1] Felice KJ. Distal myopathies. Neurol Clin. 2020;38(3):637‐659. 32703474 10.1016/j.ncl.2020.03.007

[2] Palmio J, Udd B. Myofibrillar and distal myopathies. Rev Neurol (Paris). 2016;172(10):587‐593. 27638134 10.1016/j.neurol.2016.07.019

[3] Griggs R, Vihola A, Hackman P, et al. Zaspopathy in a large classic late‐onset distal myopathy family. Brain. 2007;130(Pt 6):1477‐1484. 17337483 10.1093/brain/awm006

[4] Sjoberg G, Saavedra‐Matiz CA, Rosen DR, et al. A missense mutation in the desmin rod domain is associated with autosomal dominant distal myopathy, and exerts a dominant negative effect on filament formation. Hum Mol Genet. 1999;8(12):2191‐2198. 10545598 10.1093/hmg/8.12.2191

[5] Selcen D, Engel AG. Mutations in myotilin cause myofibrillar myopathy. Neurology. 2004;62(8):1363‐1371. 15111675 10.1212/01.wnl.0000123576.74801.75

[6] Berciano J, Gallardo E, Dominguez‐Perles R, et al. Autosomal‐dominant distal myopathy with a myotilin S55F mutation: sorting out the phenotype. J Neurol Neurosurg Psychiatry. 2008;79(2):205‐208. 17698502 10.1136/jnnp.2007.125435

[7] Duff R, Tay V, Hackman P, et al. Mutations in the N‐terminal actin‐binding domain of filamin C cause a distal myopathy. Am J Hum Genet. 2011;88(6):729‐740. 21620354 10.1016/j.ajhg.2011.04.021 PMC3113346

[8] Hackman P, Vihola A, Haravuori H, et al. Tibial muscular dystrophy is a titinopathy caused by mutations in TTN, the gene encoding the giant skeletal‐muscle protein titin. Am J Hum Genet. 2002;71(3):492‐500. 12145747 10.1086/342380 PMC379188

[9] Beggs AH, Byers TJ, Knoll JH, Boyce FM, Bruns GA, Kunkel LM. Cloning and characterization of two human skeletal muscle alpha‐actinin genes located on chromosomes 1 and 11. J Biol Chem. 1992;267(13):9281‐9288. 1339456

[10] Young P, Gautel M. The interaction of titin and alpha‐actinin is controlled by a phospholipid‐regulated intramolecular pseudoligand mechanism. EMBO J. 2000;19(23):6331‐6340. 11101506 10.1093/emboj/19.23.6331 PMC305858

[11] Ribeiro E, Pinotsis N, Ghisleni A, et al. The structure and regulation of human muscle α‐actinin. Cell. 2014;159(6):1447‐1460. 25433700 10.1016/j.cell.2014.10.056 PMC4259493

[12] Bang M‐L, Mudry RE, McElhinny AS, et al. Myopalladin, a novel 145‐kilodalton sarcomeric protein with multiple roles in Z‐disc and I‐band protein assemblies. J Cell Biol. 2001;153(2):413‐427. 11309420 10.1083/jcb.153.2.413 PMC2169455

[13] Mohapatra B, Jimenez S, Lin JH, et al. Mutations in the muscle LIM protein and alpha‐actinin‐2 genes in dilated cardiomyopathy and endocardial fibroelastosis. Mol Genet Metab. 2003;80(1–2):207‐215. 14567970 10.1016/s1096-7192(03)00142-2

[14] Chiu C, Bagnall RD, Ingles J, et al. Mutations in alpha‐actinin‐2 cause hypertrophic cardiomyopathy: a genome‐wide analysis. J Am Coll Cardiol. 2010;55(11):1127‐1135. 20022194 10.1016/j.jacc.2009.11.016

[15] Lornage X, Romero NB, Grosgogeat CA, et al. ACTN2 mutations cause "Multiple structured Core Disease" (MsCD). Acta Neuropathol. 2019;137(3):501‐519. 30701273 10.1007/s00401-019-01963-8 PMC6545377

[16] Savarese M, Palmio J, Poza JJ, et al. Actininopathy: a new muscular dystrophy caused by ACTN2 dominant mutations. Ann Neurol. 2019;85(6):899‐906. 30900782 10.1002/ana.25470

[17] Mitsuhashi S, Nonaka I, Wu S, et al. Distal myopathy in multi‐minicore disease. Intern Med. 2009;48(19):1759‐1762. 19797833 10.2169/internalmedicine.48.2425

[18] Jokela M, Tasca G, Vihola A, et al. An unusual ryanodine receptor 1 (RYR1) phenotype: mild calf‐predominant myopathy. Neurology. 2019;92(14):e1600‐e1609. 30842289 10.1212/WNL.0000000000007246

[19] Jiang B, Zhou J, Li H‐L, et al. Mutation screening in Chinese patients with familial Alzheimer's disease by whole‐exome sequencing. Neurobiol Aging. 2019;76:215.e215‐e221. 10.1016/j.neurobiolaging.2018.11.024 30598257

[20] Li LX, Dong HL, Xiao BG, Wu ZY. A novel missense mutation in peripheral myelin protein‐22 causes charcot‐marie‐tooth disease. Chin Med J (Engl). 2017;130(15):1779‐1784. 28748849 10.4103/0366-6999.211539 PMC5547828

[21] Li H‐F, Liu Z‐J, Dong H‐L, et al. Clinical features of Chinese patients with Gerstmann‐Sträussler‐Scheinker identified by targeted next‐generation sequencing. Neurobiol Aging. 2017;49:216.e211‐216.e215. 10.1016/j.neurobiolaging.2016.09.018 28340953

[22] Richards S, Aziz N, Bale S, et al. Standards and guidelines for the interpretation of sequence variants: a joint consensus recommendation of the American college of medical genetics and genomics and the association for molecular pathology. Genet Med. 2015;17(5):405‐424. 25741868 10.1038/gim.2015.30 PMC4544753

[23] Claeys KG. Congenital myopathies: an update. Dev Med Child Neurol. 2020;62(3):297‐302. 31578728 10.1111/dmcn.14365

[24] Jungbluth H, Treves S, Zorzato F, et al. Congenital myopathies: disorders of excitation‐contraction coupling and muscle contraction. Nat Rev Neurol. 2018;14(3):151‐167. 29391587 10.1038/nrneurol.2017.191

[25] Kahana E, Gratzer WB. Properties of the spectrin‐like structural element of smooth‐muscle alpha‐actinin. Cell Motil Cytoskeleton. 1991;20(3):242‐248. 1773450 10.1002/cm.970200307

[26] Fukami K, Furuhashi K, Inagaki M, Endo T, Hatano S, Takenawa T. Requirement of phosphatidylinositol 4,5‐bisphosphate for alpha‐actinin function. Nature. 1992;359(6391):150‐152. 1326084 10.1038/359150a0

[27] Nowak KJ, Ravenscroft G, Laing NG. Skeletal muscle α‐actin diseases (actinopathies): pathology and mechanisms. Acta Neuropathol. 2013;125(1):19‐32. 22825594 10.1007/s00401-012-1019-z

[28] Chauveau C, Rowell J, Ferreiro A. A rising titan: TTN review and mutation update. Hum Mutat. 2014;35(9):1046‐1059. 24980681 10.1002/humu.22611

[29] Zhou H, Yamaguchi N, Xu L, et al. Characterization of recessive RYR1 mutations in core myopathies. Hum Mol Genet. 2006;15(18):2791‐2803. 16940308 10.1093/hmg/ddl221

