# Supplementary material for: A novel frameshift ACTN2 variant causes a rare adult‐onset distal myopathy with multi‐minicores
Source: CNS Neurosci Ther. 2021 Jun 25;27(10):1198–205. doi: 10.1111/cns.13697 (PMC8446211; doi:10.1111/cns.13697)

Full unedited blot for Figure 3A-ACTN2 in the manuscript

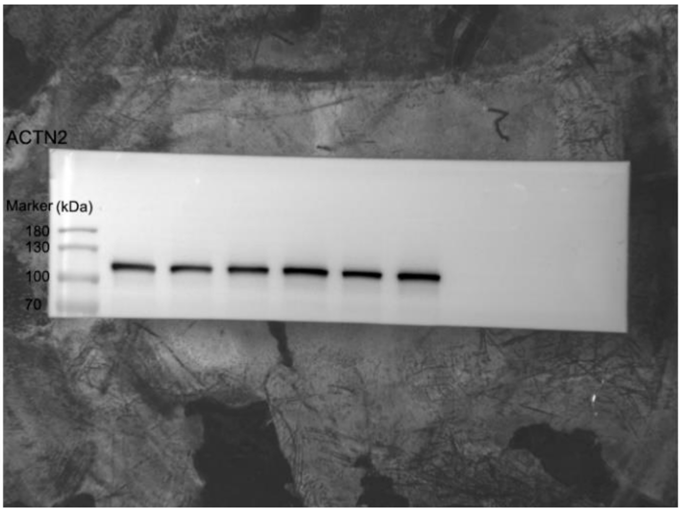

Full unedited blot for Figure 3A-ACTN2 (replicate data)

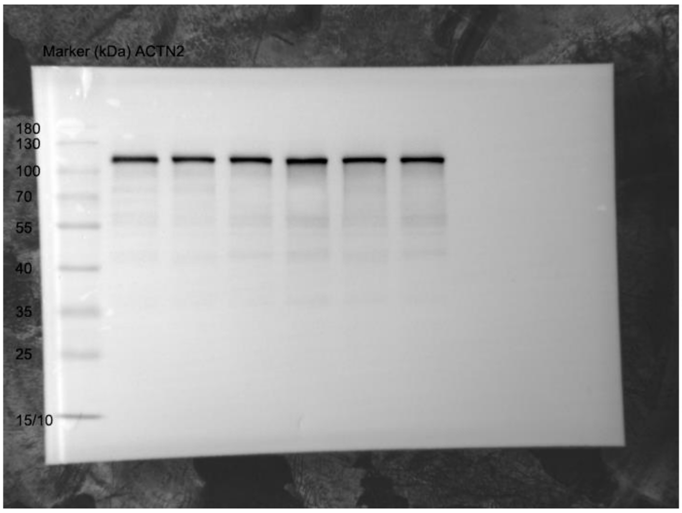

Full unedited blot for Figure 3A-ACTA1 in the manuscript

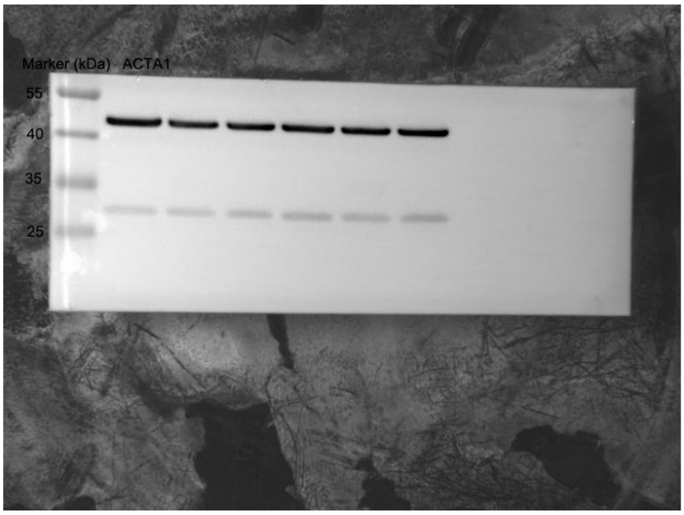

Full unedited blot for Figure 3A-ACTA1 (replicate data)

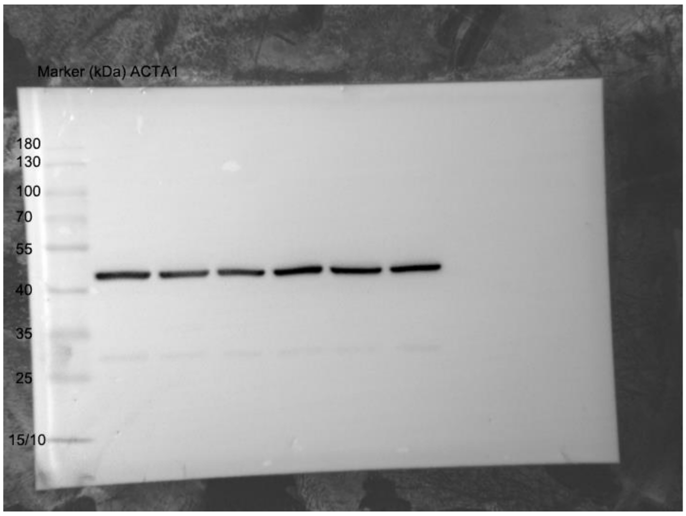

Full unedited blot for Figure 3A-GAPDH in the manuscript

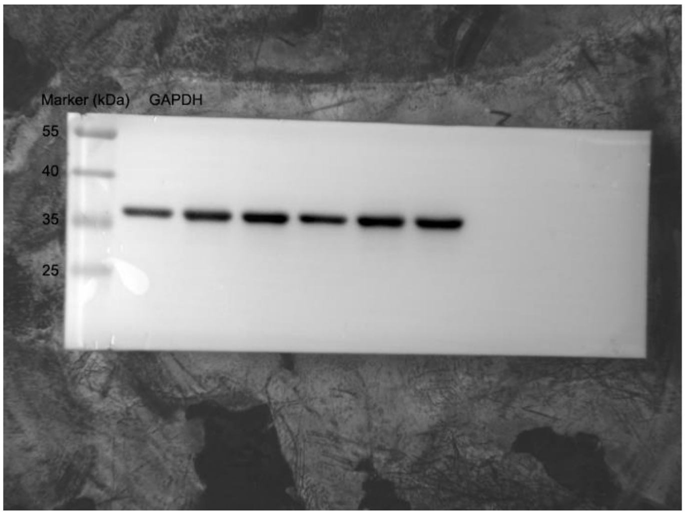

Full unedited blot for Figure 3A-GAPDH (replicate data)

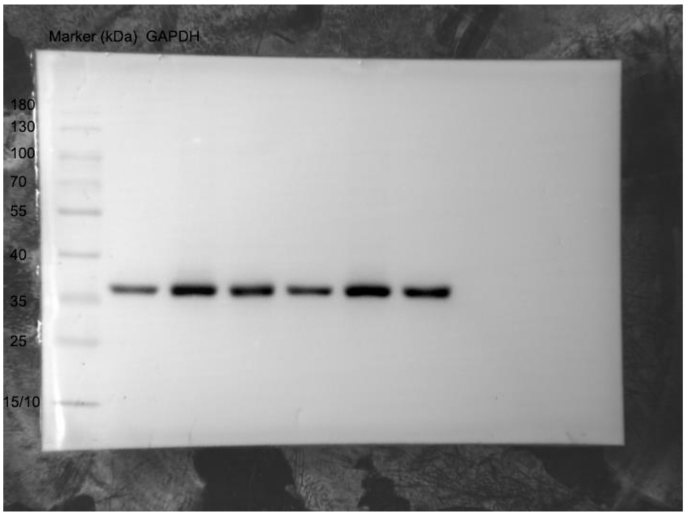

Full unedited blot for Figure 3E-Flag in the manuscript

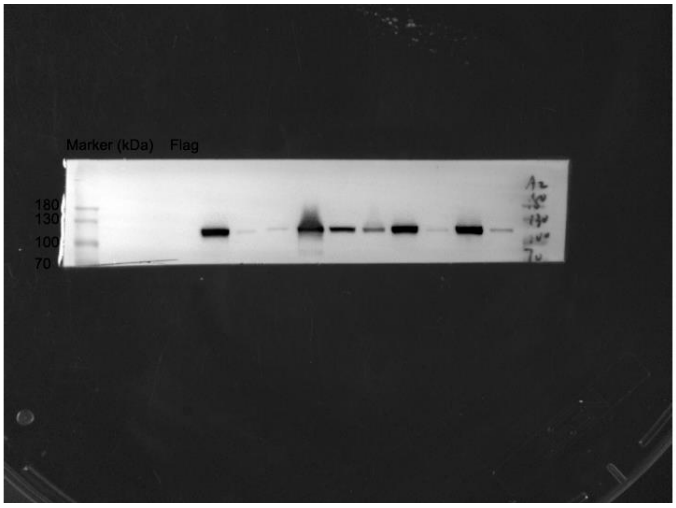

Full unedited blot for Figure 3E-Flag (replicate data)

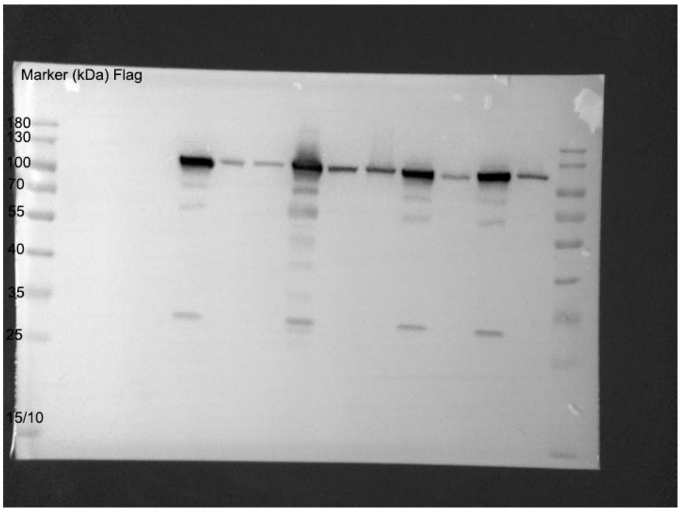

Full unedited blot for Figure 3E-Flag (replicate data)

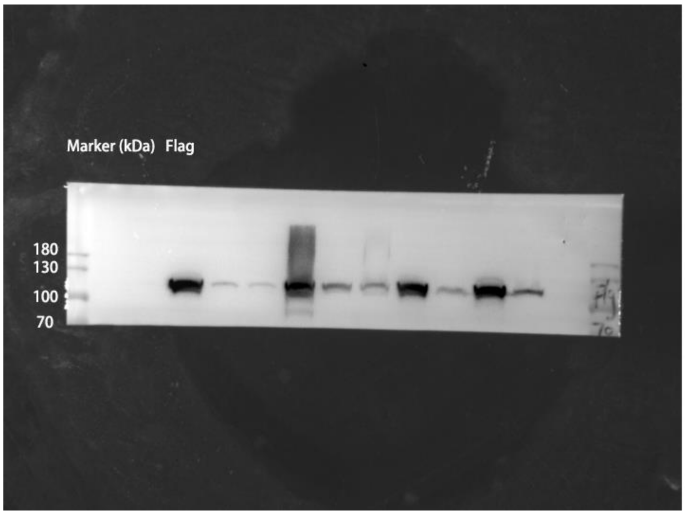

Full unedited blot for Figure 3E-β-Tubulin in the manuscript

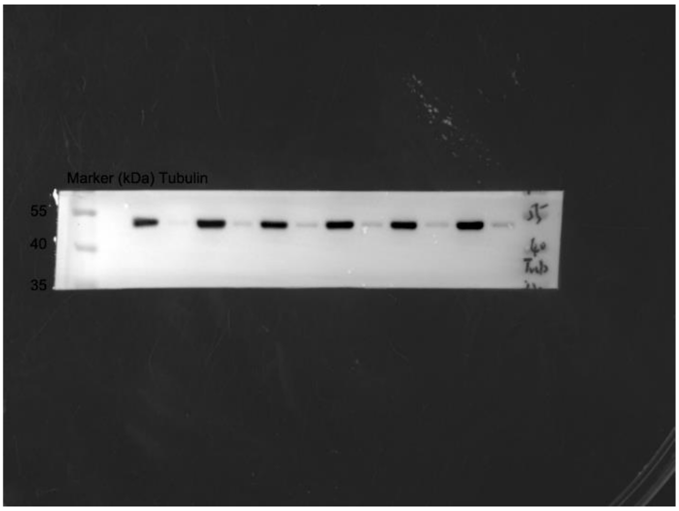

Full unedited blot for Figure 3E-β-Tubulin (replicate data)

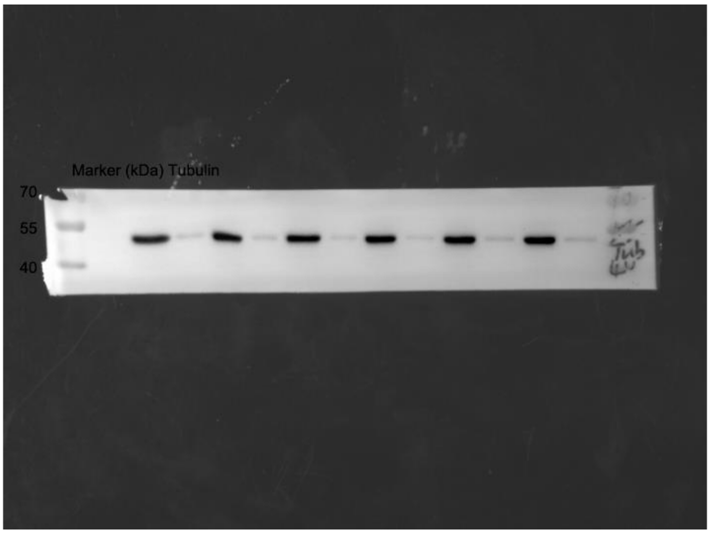

Full unedited blot for Figure 3E-β-Tubulin (replicate data)

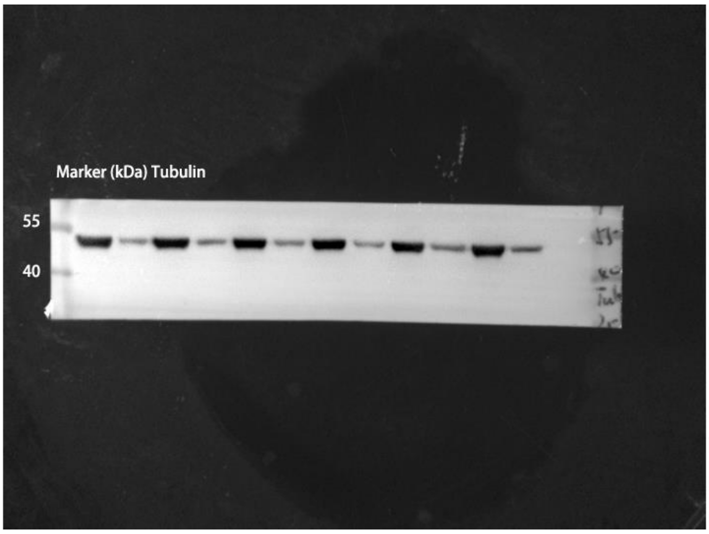

Supplement: Supplementary file 1 — Supplementary Material [file CNS-27-1198-s001.pdf]
